# Higher serum PGE2 is a predicative biomarker for postoperative delirium following elective orthopedic surgery in elderly patients

**DOI:** 10.1186/s12877-022-03367-y

**Published:** 2022-08-19

**Authors:** Meng Mao, Lei-yuan Wang, Lan-yue Zhu, Fei Wang, Ying Ding, Jian-hua Tong, Jie Sun, Qiang Sun, Mu-huo Ji

**Affiliations:** 1 Department of Anesthesiology, the Second Affiliated Hospital of Nanjing Medical University, Nanjing, China; 2 Department of Anesthesiology, the Affiliated Stomatological Hospital of Nanjing Medical University, Nanjing, Jiangsu Province China; 3 Jiangsu Province Key Laboratory of Oral Diseases, Nanjing, China; 4 Department of Anesthesiology, School of Medicine, Zhongda Hospital, Southeast University, Nanjing, China

**Keywords:** Postoperative delirium, Biomarker, PGE2, Orthopedic surgery

## Abstract

**Background:**

Postoperative delirium (POD), one of the most common complications following major surgery, imposes a heavy burden on patients and society. The objective of this exploratory study was to conduct a secondary analysis to identify whether there exist novel and reliable serum biomarkers for the prediction of POD.

**Methods:**

A total of 131 adult patients (≥ 65 years) undergoing lower extremity orthopedic surgery with were enrolled in this study. Cognitive function was assessed preoperatively with Mini-Mental State Examination (MMSE). Delirium was diagnosed according to the Confusion Assessment Method (CAM) criteria on preoperative day and postoperative days 1–3. The preoperative serum levels of a panel of 16 biochemical parameters were measured by ELISA.

**Results:**

Thirty-five patients developed POD, with an incidence of 26.7%. Patients in POD group were older (*P* = 0.001) and had lower preoperative MMSE scores (*P* = 0.001). Preoperative serum levels of prostaglandin E2 (PGE2, *P* < 0.001), S100β (*P* < 0.001), glial fibrillary acidic protein (*P* < 0.001) and neurofilament light (*P* = 0.002) in POD group were significantly increased. Logistic regression analysis showed that advanced age (OR = 1.144, 95%CI: 1.008 ~ 1.298, *P* = 0.037), higher serum neurofilament light (OR = 1.003, 95%CI: 1.000 ~ 1.005, *P* = 0.036) and PGE2 (OR = 1.031, 95%CI: 1.018 ~ 1.044, *P* < 0.001) levels were associated with the development of POD. In addition, serum level of PGE2 yielded an area under the ROC curve (AUC) of 0.897 to predict POD (*P* < 0.001), with a sensitivity of 80% and a specificity of 83.3%.

**Conclusions:**

Our study showed that higher preoperative serum PGE2 level might be a biomarker to predict the occurrence of POD in elderly patients undergoing elective orthopedic surgery.

**Trial registration:**

NCT03792373 www.clinicaltrials.gov.

## Introduction

Postoperative delirium (POD), characterized by an acute and fluctuating disturbance in awareness, cognition and attention, is one of the most common complications following major surgery [1, 2]. Although the incidence of POD in the general surgical population is 2.5–3%, it can afflict 12% to 51% of patients undergoing orthopedic surgery [3]. In high-risk patient groups, the incidence of POD is reported to be 50–70% [4]. Notably, the incidence of POD is significantly associated with increased morbidity and mortality, activities of daily living impairment, and global cognitive decline [5, 6].

The risk factors for POD are multiple and its pathophysiology remains poorly understood. Previous studies have demonstrated that advanced age, high-risk surgical procedures, cardiopulmonary comorbidities, poor functional status, preoperative fragility and cognitive impairment, pain and metabolic derangement might contribute to the occurrence of POD [7–9]. In animal studies, it has been proposed that neuroinflammation, neurohormone alterations, apoptosis, and oxidative stress may be the underlying molecular mechanism of POD [10–12]. As effective strategies exist to prevent the occurrence and development of POD [3, 4], especially in the high-risk population, there is a growing necessity to identify those patients who are vulnerable to POD.

Biomarkers not only indicate a certain pathological state, but also provide information about disease activity and progression. Although several biomarkers are reported to predict POD [10, 13], biomarkers with high sensitivity and specificity are still lacking. Therefore, we conducted this study aiming at identifying novel and reliable biomarkers for the prediction of POD. In the present study, a panel of 16 biochemical parameters in serum samples from elderly patients undergoing elective orthopedic surgery were assessed, including brain derived neurotrophic factor (BDNF), fibroblast growth factor-23 (FGF-23), irisin, C3a, C3, C5a, interleukin-17A (IL-17A), interleukin-33 (IL-33), prostaglandin E2 (PGE2), S100β, glial fibrillary acidic protein (GFAP), neurofilament light (NfL), vascular cell adhesion molecule 1 (VCAM1), E-selectin and matrix metalloprotein 9 (MMP9). Among them, BDNF, FGF-23 and irisin were selected because they are associated with neurocognitive regulation [14–16]. S100β, NfL and GFAP were implicated in neuronal injury [17, 18]. In addition, biochemical parameters associated with complement cascade (C3a, C3, C5a) and inflammatory response (IL-17A, IL-33, E-selectin, MMP9) were involved as well. These pathophysiological changes have been reported to play an essential part in the occurrence and development of POD [3, 4].

## Methods

### Study overview

**Fig. 1:**
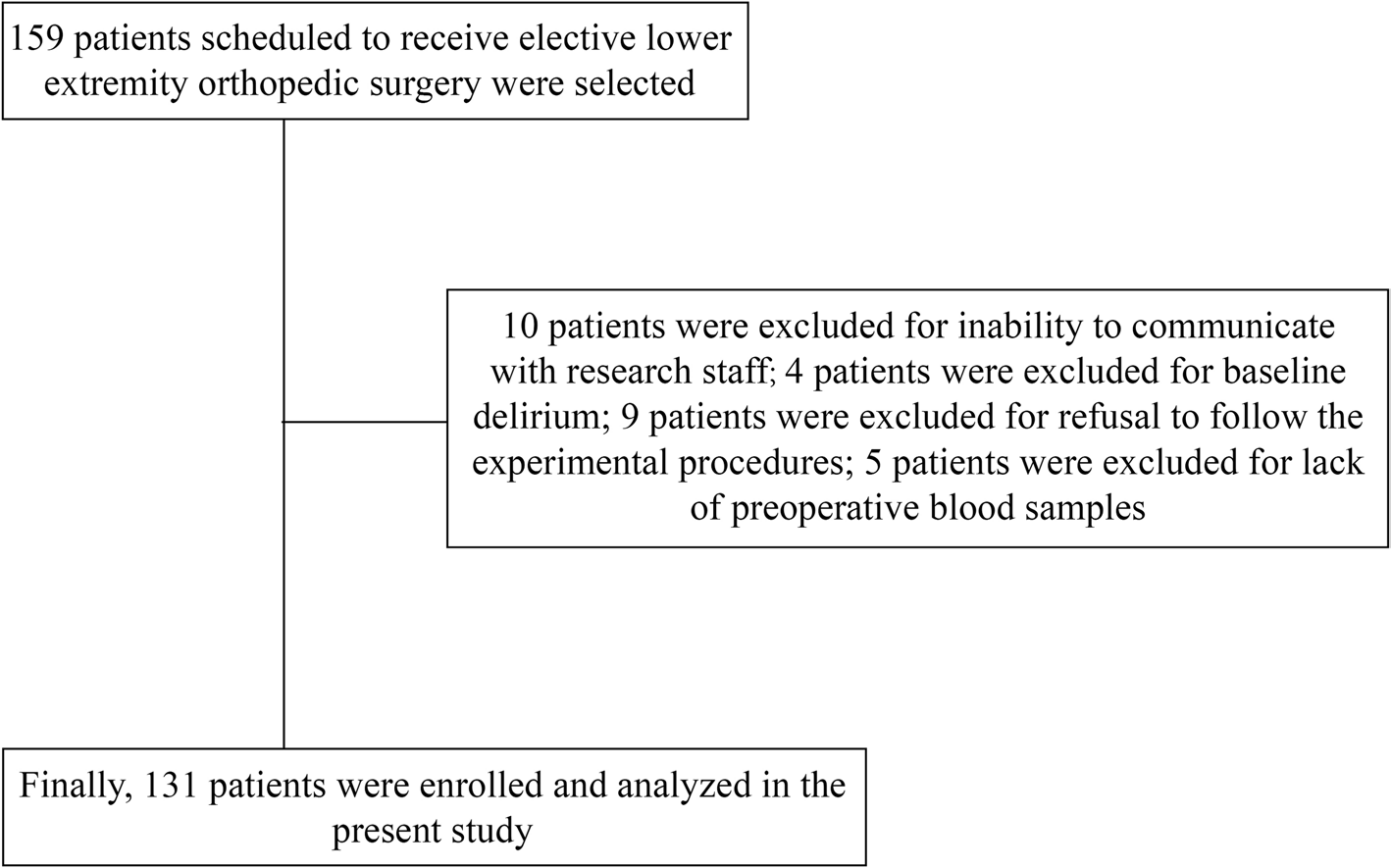
Participant flow diagram

This prospective observational study was approved by the local institutional research ethics board of Zhongda Hospital affiliated to Southeast University (2018ZDSYLL144-P01) and registered on ClinicalTrials.gov as. NCT03792373. The primary aim of this study was to determine which factors significantly influence postoperative outcome. As POD is a serious postoperative adverse complication, we included it as one of the indicators in this study. We performed this study according to the ethical principles for medical research involving human subjects detailed in the Declaration of Helsinki. Subjects were enrolled form February 1 to June 1, 2019. Written informed consent was obtained from patients prior to participation. In this secondary analysis, the inclusion criteria were age ≥ 65 years, American Society of Anesthesiologists (ASA) status I-III and undergoing elective lower extremity orthopedic surgery. Exclusion criteria were baseline delirium, refusal to follow the experimental procedures, inability to communicate with reach staff (Fig. 1).

### Delirium and cognition assessment

Mini-mental state examination (MMSE) was used to assess the preoperative cognitive function of participants on the day before surgery. The Confusion Assessment Method (CAM) was utilized for the evaluation of delirium, which comprises four clinical features: (a) acute onset of cognitive changes with a fluctuating course, (b) inattention, (c) disorganized thinking, and (d) altered level of consciousness. The diagnosis of delirium was based on the presence of (a) and (b) plus either (c) or (d). For baseline delirium assessment, participants enrolled were evaluated by a well-trained anesthesiologist on the day before surgery. For POD diagnosis, participants enrolled were evaluated by the same anesthesiologist twice daily on postoperative days 1–3.

### Biochemical measurements

Patients had blood draws in the morning on the day before surgery. The collected serum samples were stored at -80℃ for the later detection. The serum levels of 16 biochemical parameters were measured by enzyme linked immunosorbent assay (ELISA, CUSABIO, Wuhan, China), including BDNF, FGF-23, irisin, C3a, C3, C5a, IL-17A, IL-33, PGE2, S100β, GFAP, NfL, VCAM1, E-selectin and MMP9.

### Statistical analysis

This was a secondary analysis of data from a prospective observational study. Statistical analysis was performed using the SPSS version 25.0 software (SPSS, Chicago, IL). Normally distributed quantitative data were presented as mean and SD and were analyzed with independent-sample t-test, while non-normally distributed quantitative data were presented as median and interquartile range (IQR) and were analyzed with Mann–Whitney U test. Categorical data were analyzed with χ^2^ test or Fisher exact test. The effect size of all significant group differences was calculated with the formula Cohen’s d = (M_A_- M_B_) / σ or *r* = Z /$\sqrt{N}$ depending on the distribution of the data [19]. The risk factors for POD were evaluated by multivariate logistic regression analysis, in which variables with *P* values less than 0.05 were included. The prediction of serum biomarkers for POD was analyzed by using receiver operating characteristic (ROC) curve. *P* < 0.05 was regarded as statistically significant.

## Results

### Patient characteristics at baseline

A total of 131 patients were finally included in this study, and 35 of them were diagnosed with POD (35/131, 26.7%). The baseline characteristics in POD and non-POD groups were presented in Table 1. No significant difference was observed between the two groups in terms of sex, BMI, ASA status, medical comorbidities (hypertension, diabetes mellitus, coronary artery disease, history of stroke, chronic pulmonary disease), duration of surgery, blood loss (all *P* > 0.05). Patients in POD group were older (*P* = 0.001) and had lower preoperative MMSE scores (*P* = 0.001) compared with those in non-POD group.

**Table 1:** Preoperative characteristics of patients with or without POD

Characteristics	POD group (*n* = 35)	Non-POD group (*n* = 96)	^a^*P* value	effect size
Sex (n, male/female)	15/20	39/57	0.818	/
Age (year)	77(70 ~ 82)	72(67 ~ 76)	0.001	0.30
ASA status (I/II/III)	2/22/11	8/73/15	0.169	/
BMI (kg/m^2^)	21.88(19.58 ~ 25.40)	23.50(22.04 ~ 26.38)	0.058	/
Preoperative MMSE	22(18 ~ 27)	26(23 ~ 29)	0.001	0.30
Medical comorbidities (n)				
Hypertension	29(82.86%)	78(81.25%)	0.833	/
Diabetes mellitus	13(37.14%)	30(31.25%)	0.525	/
Coronary artery disease	11(31.43%)	27(28.13%)	0.712	/
History of stroke	10(28.57%)	19(19.79%)	0.284	/
Chronic pulmonary disease	6(17.14%)	16(16.67%)	0.949	/
Duration of surgery (min)	85(75 ~ 95)	80(70 ~ 90)	0.454	/
Blood loss(ml)	250(200 ~ 300)	250(200 ~ 350)	0.815	/

*POD* Postoperative delirium, *ASA* American Society of Anesthesiologists, *BMI* Body Mass Index, *MMSE* Mini-Mental State Examination

^a^Continuous normally distributed variables (data presented as mean and SD) were analyzed with independent-sample t-test. Continuous non-normally distributed variables (data presented as median and IQR) were analyzed with Mann–Whitney U test. Categorical data were analyzed with χ^2^ test or Fisher exact test (*n* = 131)

### Differences in the levels of preoperative biochemical parameters between patients with and without POD

As shown in Table 2, serum levels of PGE2 (*P* < 0.001), S100β (*P* < 0.001), GFAP (*P* < 0.001), and NfL (*P* = 0.002) were significantly increased in POD group. However, no significant difference was observed in BDNF, FGF-23, irisin, C3a, C3, C5a, IL-17A, IL-33, VCAM1, E-selectin and MMP9. (*P* > 0.05).

**Table 2:** Intergroup comparisons of the preoperative serum biochemical parameters

Parameters	POD group (*n* = 35)	Non-POD group (*n* = 96)	^a^*P* value	effect size
BDNF (ng/ml)	0.43(0.38 ~ 0.54)	0.48(0.41 ~ 0.63)	0.109	/
FGF-23 (pg/ml)	504.07(456.31 ~ 577.63)	513.83(466.68 ~ 588.38)	0.894	/
Irisin (ng/ml)	47.64(18.94 ~ 74.45)	63.01(26.67 ~ 115.62)	0.070	/
IL-17A (pg/ml)	6.00(5.71 ~ 6.61)	6.02(5.58 ~ 6.69)	0.320	/
IL-33 (pg/ml)	11.50(5.35 ~ 33.07)	16.21(5.13 ~ 185.00)	0.353	/
C3a (ng/ml)	504.07(456.31 ~ 577.63)	513.83(466.68 ~ 588.38)	0.894	/
C3 (μg/ml)	264.87(136.56 ~ 297.21)	207.81(103.38 ~ 271.51)	0.065	/
C5a (ng/ml)	102.57(52.33 ~ 165.90)	85.77(52.71 ~ 141.67)	0.297	/
iNOS (IU/ml)	8.81(6.21 ~ 13.6)	8.54(6.07 ~ 12.16)	0.274	/
PGE2 (pg/ml)	181.23(126.08 ~ 235.65)	61.63(39.96 ~ 105.65)	< 0.001	0.61
S100β (pg/ml)	199.22(183.52 ~ 283.19)	181.36(170.91 ~ 207.67)	< 0.001	0.33
GFAP (ng/ml)	11.76(7.77 ~ 14.61)	7.38(5.84 ~ 10.29)	< 0.001	0.36
NfL (ng/ml)	1.47 ± 0.2960	1.29 ± 0.2961	0.002	0.60
VCAM1(ng/ml)	691.11 ± 263.50	652.18 ± 196.83	0.364	/
E-selectin (ng/ml)	2.95(2.14 ~ 3.99)	2.79(1.98 ~ 4.26)	0.718	/
MMP9 (ng/ml)	4.00(1.37 ~ 5.32)	3.36(1.71 ~ 9.68)	0.649	/

*POD* Postoperative delirium, *BDNF* Brain derived neurotrophic factor, *FGF-23* Fibroblast growth factor 23, *IL* Interleukin, *iNOS* Inducible nitric oxide synthase, *PGE2* Prostaglandin E2, *GFAP* Glial fibrillary acidic protein, *NfL* Neurofilament light, *VCAM* Vascular cell adhesion molecule, *MMP* Matrix metalloprotein

^a^Continuous normally distributed variables (data presented as mean and SD) were analyzed with independent-sample t-test. Continuous non-normally distributed variables (data presented as median and IQR) were analyzed with Mann–Whitney U test (*n* = 131)

### Preoperative biomarker associations with POD

Then, a total of six clinical and biochemical variables with significant difference were assessed as potential predictors of POD by using multivariate logistic regression. As illustrated in Table 3, regression analysis showed that advanced age (OR = 1.144, 95%CI: 1.008 ~ 1.298, *P* = 0.037), higher serum NfL (OR = 1.003, 95%CI: 1.000 ~ 1.005, *P* = 0.036) and PGE2 (OR = 1.031, 95% CI: 1.018 ~ 1.044, *P* < 0.001) levels were predicative factors for POD.

**Table 3:** Logistics regression analysis of POD risk factors

Variables	OR	95% CI	*P* value
Age	1.144	1.008 ~ 1.298	0.037
Preoperative MMSE	0.922	0.810 ~ 1.049	0.217
PGE2	1.031	1.018 ~ 1.044	< 0.001
S100β	1.002	0.997 ~ 1.008	0.456
NfL	1.003	1.000 ~ 1.005	0.036
GFAP	1.021	0.967 ~ 1.078	0.452

*POD* Postoperative delirium, *OR* Odds ratio, *CI* Credibility interval, *MMSE* Mini-Mental State Examination, *PGE2* Prostaglandin E2, *NfL* Neurofilament light, *VCAM* Vascular cell adhesion molecule, *GFAP* Glial fibrillary acidic protein; (*n* = 131)

### The predicative performance of age, NfL and PGE2 for POD

**Fig. 2:**
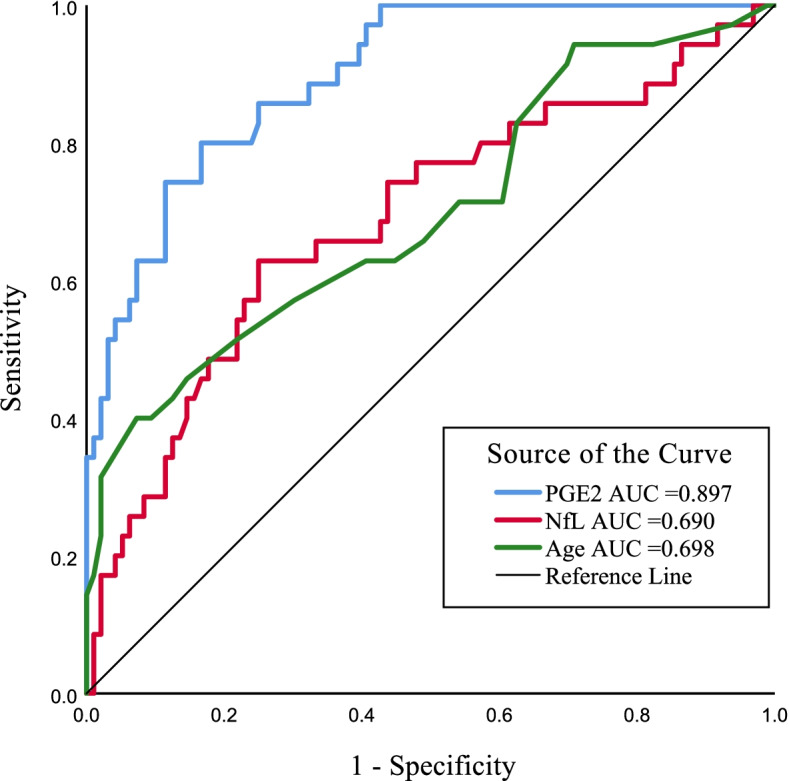
ROC curve analysis of age, preoperative serum NfL and PEC2 levels for distinguishing POD. (*n* = 131). ROC: receiver operating characteristic; NfL: neurofilament light; PGE2: prostaglandin E2; POD: postoperative delirium

The predicative performance of age, serum NfL and PGE2 levels for POD was identified by plotting ROC curve. As shown in Fig. 2, preoperative serum level of PGE2 had a good performance in predicting POD with the area under the ROC curve (AUC) value of 0.897 and 95% CI of 0.842–0.952 (*P* < 0.001). The cut-off point of PGE2 level to predict POD was ≥ 124.1 ng/L, while the sensitivity and specificity for PGE2 were 80% and 83.3%, respectively. In addition, advanced age (AUC = 0.698, 95%CI: 0.590 ~ 0.806, *P* = 0.001), and higher NfL level (AUC = 0.690, 95%CI: 0.581 ~ 0.798, *P* = 0.001) could also discriminated patients with POD.

## Discussion

Our study showed that preoperative serum level of PGE2 was closely associated with the development of POD in elderly patients undergoing elective orthopedic surgery. Thus, preoperative PGE2 could serve as a new serum biomarker to identify patients at risk for developing POD. To the best of our knowledge, this is the first study to link preoperative PGE2 level with POD following elective orthopedic surgery.

The incidence of POD was 26.7% in our study, which was similar to 27% in orthopedic surgery reported by a systematic review [20], and was also comparable with 20.4% in the aged patients undergoing similar type of surgery in a recent study [21]. It is widely accepted that the occurrence of POD was resulted from the complicated interactions of multiple predisposing factors. Among the known risk factors for POD, advanced age and pre-existing dementia or cognitive impairment have been considered as the major risk factors [3]. Consistently, our study showed advanced age was an independent risk factor of POD, and patients with POD were prone to have poor preoperative cognition as reflected by MMSE. Although delirium is significantly associated with adverse outcomes, fortunately, it is preventable and treatable [3]. Currently, non-pharmacological multicomponent intervention is thought to be the most effective prevention strategy, which mainly includes the Hospital Elder Life Program and proactive old age medicine consultation [22–24]. In addition, the fundamental aim of all preoperative screening instruments, whether scale- or biomarker-based, is to identify those vulnerable patients, who then can benefit from optimized perioperative evaluation, monitoring and management. Thus, identifying high-risk patients is an important step in successfully reducing the risk for POD.

Despite various mechanisms have been implicated in the development of POD [12, 25], neuroinflammation and altered neurotransmitters are thought to be the two leading hypotheses [26]. For neuroinflammation, both preoperative and postoperative elevation of peripheral C-reactive protein and IL-6 were reported to be associated with higher risks of POD [27]. The underlying mechanism may be that peripheral inflammation causes the breakdown of the blood–brain barrier, leading to neuroinflammation, which in turn results in synaptic loss, neuronal death, and neurogenesis damage [28]. On the other hand, studies have showed alteration of neurotransmitters in the process of POD, including acetylcholine, glutamate, gamma-aminobutyric acid, dopamine, and serotonin systems, of which deficient choline or excessive dopamine, or both, are the most frequently linked to delirium [3, 4, 26].

In the present study, we assessed a panel of 16 parameters implicated in inflammatory response, neurotransmitter disturbance, complement cascade, and neuronal injury in serum samples from elderly patients undergoing elective orthopedic surgery. Importantly, we showed that serum level of preoperative PGE2 was significantly increased and appeared to be a potential biomarker of POD after elective orthopedic surgery. Indeed, PGE2 is reported to be an important modulator of inflammation, which is of particular interest due to its relatively high concentrations in the brain compared with other prostaglandins [29]. PGE2 works through four distinct G protein-coupled E-prostanoid receptors, EP1 to EP4, and a growing number of studies suggest that PGE2 exerts neurotoxic effects by binding to EP1, EP2 or EP3 receptors in models of chronic neurodegenerative disease like Alzheimer’s disease (AD) and Parkinson’s disease [29, 30]. In aged mice, blockade of peripheral PGE2 signaling is sufficient to reverse cognitive decline associated with aging [31]. Moreover, the cerebrospinal fluid concentration of PGE2 is elevated in both probable AD patients and in AD patients with memory decline [32, 33]. Strikingly, subsequent analysis of several studies indicated that chronic non-steroid anti-inflammatory drug use was associated with lower risk of AD in normal aging people [29, 34]. However, there are a number of studies suggesting that PGE2 had a role in synaptic plasticity and neuroprotection by binding EP4 receptors [35, 36]. Therefore, the role of PGE2 is complex and whether PGE2 inhibitor can be used as a prevention or treatment target for POD in elderly patients needs further research.

Our study also showed that NfL was also an independent risk factor for POD. As one of the neuroaxonal injury markers, NfL has been reported to be elevated in multiple conditions including stroke, traumatic brain injury, multiple sclerosis, AD and frontotemporal dementia [37]. Recently, a clinical study by Tamara and colleagues showed that patients with the highest preoperative NfL levels were more likely to develop POD, which is consistent with our finding [38]. Additionally, the increased serum levels of S100β and GFAP were observed in POD group. S100β, a calcium binding protein, has different effects during different stages of human development. In the first three months of life, S100β exerts neurotrophic effects on the central nervous system (CNS) [39]. However, elevated S100β levels at other points in life mostly correlate with blood–brain barrier injury, which has been observed in cerebrovascular and neurodegenerative diseases [40, 41]. S100β has been reported as a potential biomarker for delirium, but its particular relationship with POD remains controversial [42, 43]. GFAP, an intermediate filament protein, is mainly expressed in astrocytes and its plasma level has been proposed as a biomarker for nervous system injury in traumatic brain injury and stroke [44, 45]. Overall, these increased preoperative serum levels of S100β and GFAP reflect pre-existing brain function impairment in patients with POD, which makes them more susceptible to brain injury in response to surgical trauma.

A few limitations should be noted in our study. Firstly, despite suitable for identifying biomarker, this clinical study involved a single-center, single-surgery procedure and was conducted in a relatively small sample, which may produce biases unintentionally, thus further studies in larger sample size are required. Moreover, detecting PGE2 levels postoperatively would be more informative to determine the relationship between PGE2 and POD, which merits further investigation. Finally, the information about the perioperative use of non-steroid anti-inflammatory drug was not collected, which might have an effect on our study results and should be considered in future study.

## Conclusions

Our study showed that preoperative serum PGE2 levels might be a biomarker to predict the occurrence of POD in elderly patients undergoing elective orthopedic surgery. Meanwhile, this finding may provide a target for possible interventions to prevent POD. However, further studies in different surgical populations are required to confirm our results.

## Data Availability

The datasets used and/or analysed during the current study are available from the corresponding author on reasonable request.

## Abbreviations

AD: Alzheimer’s disease
ASA: American Society of Anesthesiologists
AUC: Area under the ROC curve
BDNF: Brain derived neurotrophic factor
BMI: Body mass index
CAM: The Confusion Assessment Method
CI: Confidence interval
CSF: Cerebrospinal fluid
ELISA: Enzyme linked immunosorbent assay
FGF-23: Fibroblast growth factor
GFAP: Glial fibrillary acidic protein
IL: Interleukin
IQR: Interquartile range
MMP9: Matrix metalloprotein 9
MMSE: Mini-Mental State Examination
NfL: Neurofilament light
OR: Odds ratio
PGE2: Prostaglandin E2
POD: Postoperative delirium
ROC: Receiver operating characteristic
VCAM1: Vascular cell adhesion molecule 1

## References

[1] Igwe EO, Nealon J, Mohammed M, Hickey B, Chou KR, Chen KH (2020). Multi-disciplinary and pharmacological interventions to reduce post-operative delirium in elderly patients: A systematic review and meta-analysis. J Clin Anesth.

[2] Boone MD, Sites B, von Recklinghausen FM, Mueller A, Taenzer AH, Shaefi S (2020). Economic Burden of Postoperative Neurocognitive Disorders Among US Medicare Patients. JAMA Netw Open.

[3] Inouye SK, Westendorp RG, Saczynski JS (2014). Delirium in elderly people. Lancet.

[4] Jin Z, Hu J, Ma D (2020). Postoperative delirium: perioperative assessment, risk reduction, and management. Br J Anaesth.

[5] Pedemonte JC, Sun H, Franco-Garcia E, Zhou C, Heng M, Quraishi SA (2021). Postoperative delirium mediates 180-day mortality in orthopaedic trauma patients. Br J Anaesth.

[6] Shi Z, Mei X, Li C, Chen Y, Zheng H, Wu Y (2019). Postoperative Delirium Is Associated with Long-term Decline in Activities of Daily Living. Anesthesiology.

[7] Harris MJ, Brovman EY, Urman RD (2019). Clinical predictors of postoperative delirium, functional status, and mortality in geriatric patients undergoing non-elective surgery for hip fracture. J Clin Anesth.

[8] Susano MJ, Grasfield RH, Friese M, Rosner B, Crosby G, Bader AM (2020). Brief Preoperative Screening for Frailty and Cognitive Impairment Predicts Delirium after Spine Surgery. Anesthesiology.

[9] Iamaroon A, Wongviriyawong T, Sura-Arunsumrit P, Wiwatnodom N, Rewuri N, Chaiwat O (2020). Incidence of and risk factors for postoperative delirium in older adult patients undergoing noncardiac surgery: a prospective study. BMC Geriatr.

[10] Shen H, Shao Y, Chen J, Guo J (2016). Insulin-Like Growth Factor-1, a Potential Predicative Biomarker for Postoperative Delirium Among Elderly Patients with Open Abdominal Surgery. Curr Pharm Des.

[11] Noah AM, Almghairbi D, Evley R, Moppett IK (2021). Preoperative inflammatory mediators and postoperative delirium: systematic review and meta-analysis. Br J Anaesth.

[12] Lopez MG, Hughes CG, DeMatteo A, O’Neal JB, McNeil JB, Shotwell MS, et al. Intraoperative Oxidative Damage and Delirium after Cardiac Surgery. Anesthesiology. 2020;132(3):551–61. 10.1097/ALN.0000000000003016 PMC7015795 31770146

[13] Lv XC, Lin Y, Wu QS, Wang L, Hou YT, Dong Y (2021). Plasma interleukin-6 is a potential predictive biomarker for postoperative delirium among acute type a aortic dissection patients treated with open surgical repair. J Cardiothorac Surg.

[14] Hensel N, Schon A, Konen T, Lubben V, Forthmann B, Baron O (2016). Fibroblast growth factor 23 signaling in hippocampal cells: impact on neuronal morphology and synaptic density. J Neurochem.

[15] Lu B, Nagappan G, Lu Y (2014). BDNF and synaptic plasticity, cognitive function, and dysfunction. Handb Exp Pharmacol.

[16] Islam MR, Valaris S, Young MF, Haley EB, Luo R, Bond SF (2021). Exercise hormone irisin is a critical regulator of cognitive function. Nat Metab.

[17] Goncalves CA, Leite MC, Nardin P (2008). Biological and methodological features of the measurement of S100B, a putative marker of brain injury. Clin Biochem.

[18] Shahim P, Politis A, van der Merwe A, Moore B, Ekanayake V, Lippa SM (2020). Time course and diagnostic utility of NfL, tau, GFAP, and UCH-L1 in subacute and chronic TBI. Neurology.

[19] Fritz CO, Morris PE, Richler JJ (2012). Effect size estimates: current use, calculations, and interpretation. J Exp Psychol Gen.

[20] Ho MH, Nealon J, Igwe E, Traynor V, Chang HR, Chen KH (2021). Postoperative Delirium in Older Patients: A Systematic Review of Assessment and Incidence of Postoperative Delirium. Worldviews Evid Based Nurs.

[21] Sun J, Zhang Q, Lin B, He M, Pang Y, Liang Q (2021). Association Between Postoperative Long-Term Heart Rate Variability and Postoperative Delirium in Elderly Patients Undergoing Orthopedic Surgery: A Prospective Cohort Study. Front Aging Neurosci.

[22] Durst J, Wilson D (2020). Effects of protocol on prevention of delirium in hospitalized hip fracture patients: A quality improvement project. Int J Orthop Trauma Nurs.

[23] O’Mahony R, Murthy L, Akunne A, Young J. Synopsis of the National Institute for Health and Clinical Excellence guideline for prevention of delirium. Ann Intern Med. 2011;154(11):746–51. 10.7326/0003-4819-154-11-201106070-00006 21646557

[24] Todd OM, Teale EA (2017). Delirium: a guide for the general physician. Clin Med (Lond).

[25] Wyrobek J, LaFlam A, Max L, Tian J, Neufeld KJ, Kebaish KM (2017). Association of intraoperative changes in brain-derived neurotrophic factor and postoperative delirium in older adults. Br J Anaesth.

[26] Oh ST, Park JY (2019). Postoperative delirium. Korean J Anesthesiol.

[27] Liu X, Yu Y, Zhu S (2018). Inflammatory markers in postoperative delirium (POD) and cognitive dysfunction (POCD): A meta-analysis of observational studies. PLoS ONE.

[28] Alam A, Hana Z, Jin Z, Suen KC, Ma D (2018). Surgery, neuroinflammation and cognitive impairment. EBioMedicine.

[29] Woodling NS, Andreasson KI. Untangling the Web: Toxic and Protective Effects of Neuroinflammation and PGE2 Signaling in Alzheimer’s Disease. ACS Chem Neurosci. 2016;7(4):454–63. 10.1021/acschemneuro.6b00016 PMC5239037 26979823

[30] Andreasson K (2010). Emerging roles of PGE2 receptors in models of neurological disease. Prostaglandins Other Lipid Mediat.

[31] Minhas PS, Latif-Hernandez A, McReynolds MR, Durairaj AS, Wang Q, Rubin A (2021). Restoring metabolism of myeloid cells reverses cognitive decline in ageing. Nature.

[32] Combrinck M, Williams J, De Berardinis MA, Warden D, Puopolo M, Smith AD, et al. Levels of CSF prostaglandin E2, cognitive decline, and survival in Alzheimer’s disease. J Neurol Neurosurg Psychiatry. 2006;77(1):85–8. 10.1136/jnnp.2005.063131 PMC2117387 15944180

[33] Montine TJ, Sidell KR, Crews BC, Markesbery WR, Marnett LJ, Roberts LN (1999). Elevated CSF prostaglandin E2 levels in patients with probable AD. Neurology.

[34] Cunningham C, Skelly DT (2012). Non-steroidal anti-inflammatory drugs and cognitive function: are prostaglandins at the heart of cognitive impairment in dementia and delirium?. J Neuroimmune Pharmacol.

[35] Shi J, Johansson J, Woodling NS, Wang Q, Montine TJ, Andreasson K (2010). The prostaglandin E2 E-prostanoid 4 receptor exerts anti-inflammatory effects in brain innate immunity. J Immunol.

[36] Sang N, Zhang J, Marcheselli V, Bazan NG, Chen C (2005). Postsynaptically synthesized prostaglandin E2 (PGE2) modulates hippocampal synaptic transmission via a presynaptic PGE2 EP2 receptor. J Neurosci.

[37] Gaetani L, Blennow K, Calabresi P, Di Filippo M, Parnetti L, Zetterberg H (2019). Neurofilament light chain as a biomarker in neurological disorders. J Neurol Neurosurg Psychiatry.

[38] Fong TG, Vasunilashorn SM, Ngo L, Libermann TA, Dillon ST, Schmitt EM (2020). Association of Plasma Neurofilament Light with Postoperative Delirium. Ann Neurol.

[39] Bouvier D, Duret T, Rouzaire P, Jabaudon M, Rouzaire M, Nourrisson C (2016). Preanalytical, analytical, gestational and pediatric aspects of the S100B immuno-assays. Clin Chem Lab Med.

[40] Chong ZZ, Changyaleket B, Xu H, Dull RO, Schwartz DE (2016). Identifying S100B as a Biomarker and a Therapeutic Target For Brain Injury and Multiple Diseases. Curr Med Chem.

[41] D’Cunha NM, McKune AJ, Panagiotakos DB, Georgousopoulou EN, Thomas J, Mellor DD, et al. Evaluation of dietary and lifestyle changes as modifiers of S100beta levels in Alzheimer’s disease. Nutr Neurosci. 2019;22(1):1–18. 10.1080/1028415X.2017.1349032 28696163

[42] Khan BA, Perkins AJ, Prasad NK, Shekhar A, Campbell NL, Gao S (2020). Biomarkers of Delirium Duration and Delirium Severity in the ICU. Crit Care Med.

[43] Zhang X, Lyu Y, Wang D (2020). S100beta as a potential biomarker of incident delirium: a systematic review and meta-analysis. Minerva Anestesiol.

[44] Wunderlich MT, Wallesch CW, Goertler M (2006). Release of glial fibrillary acidic protein is related to the neurovascular status in acute ischemic stroke. Eur J Neurol.

[45] Mondello S, Guedes VA, Lai C, Czeiter E, Amrein K, Kobeissy F (2020). Circulating Brain Injury Exosomal Proteins following Moderate-To-Severe Traumatic Brain Injury: Temporal Profile, Outcome Prediction and Therapy Implications. Cells.

