# Correction: Higher serum PGE2 is a predicative biomarker for postoperative delirium following elective orthopedic surgery in elderly patients

**DOI:** 10.1186/s12877-022-03401-z

**Published:** 2022-09-07

**Authors:** Meng Mao, Lei-yuan Wang, Lan-yue Zhu, Fei Wang, Ying Ding, Jian-hua Tong, Jie Sun, Qiang Sun, Mu-huo Ji

**Affiliations:** 1 Department of Anesthesiology, the Affiliated Stomatological Hospital of Nanjing Medical University, Nanjing, Jiangsu Province China; 2 Department of Anesthesiology, the Second Affiliated Hospital of Nanjing Medical University, Nanjing, China; 3 Department of Anesthesiology, Zhongda Hospital, School of Medicine, Southeast University, Nanjing, China; 4 Jiangsu Province Key Laboratory of Oral Diseases, Nanjing, China

**Correction: BMC Geriatr 22, 685 (2022)**

**https://doi.org/10.1186/s12877-022-03367-y**

After publication of this article [1], the authors reported that authors are partly linked to the wrong affiliation. Correct is:

Meng Mao1,4, Lei-yuan Wang2, Lan-yue Zhu3, Fei Wang2, Ying Ding2, Jian-hua Tong2, Jie Sun3, Qiang Sun1,4, Mu-huo Ji2

1Department of Anesthesiology, the Affiliated Stomatological Hospital of Nanjing Medical University, Nanjing, Jiangsu Province, China.

2Department of Anesthesiology, the Second Affiliated Hospital of Nanjing Medical University, Nanjing, China.

3Department of Anesthesiology, Zhongda Hospital, School of Medicine, Southeast University, Nanjing, China.

4Jiangsu Province Key Laboratory of Oral Diseases. Nanjing, China.

The original article [1] has been corrected.
